# Evaluation of nipple aspirate fluid as a diagnostic tool for early detection of breast cancer

**DOI:** 10.1186/s12014-017-9179-4

**Published:** 2018-01-11

**Authors:** Sadr-ul Shaheed, Catherine Tait, Kyriacos Kyriacou, Richard Linforth, Mohamed Salhab, Chris Sutton

**Affiliations:** 1 Institute of Cancer Therapeutics, University of Bradford, Bradford, UK; 2 Bradford Teaching Hospitals NHS Trust, Bradford, UK; 3 The Cyprus School of Molecular Medicine, The Cyprus Institute of Neurology and Genetics, Nicosia, Cyprus

**Keywords:** Breast cancer, Biomarkers, Proteomics, Liquid biopsy, Nipple aspirate fluid (NAF)

## Abstract

There has been tremendous progress in detection of breast cancer in postmenopausal women, resulting in two-thirds of women surviving more than 20 years after treatment. However, breast cancer remains the leading cause of cancer-related deaths in premenopausal women. Breast cancer is increasing in younger women due to changes in life-style as well as those at high risk as carriers of mutations in high-penetrance genes. Premenopausal women with breast cancer are more likely to be diagnosed with aggressive tumours and therefore have a lower survival rate. Mammography plays an important role in detecting breast cancer in postmenopausal women, but is considerably less sensitive in younger women. Imaging techniques, such as contrast-enhanced MRI improve sensitivity, but as with all imaging approaches, cannot differentiate between benign and malignant growths. Hence, current well-established detection methods are falling short of providing adequate safety, convenience, sensitivity and specificity for premenopausal women on a global level, necessitating the exploration of new methods. In order to detect and prevent the disease in high risk women as early as possible, methods that require more frequent monitoring need to be developed. The emergence of “omics” strategies over the last 20 years, enabling the characterisation and understanding of breast cancer at the molecular level, are providing the potential for long term, longitudinal monitoring of the disease. Tissue and serum biomarkers for breast cancer stratification, diagnosis and predictive outcome have emerged, but have not successfully translated into clinical screening for early detection of the disease. The use of breast-specific liquid biopsies, such as nipple aspirate fluid (NAF), a natural secretion produced by breast epithelial cells, can be collected non-invasively for biomarker profiling. As we move towards an age of active surveillance, home-based liquid biopsy collection kits are increasingly being applied and these could provide a paradigm shift where NAF biomarker profiling is used for routine breast health monitoring. The current status of established and newly emerging imaging techniques for early detection of breast cancer and the potential for alternative biomarker screening of liquid biopsies, particularly those applied to high-risk, premenopausal women, will be reviewed.

## Background

Breast cancer is a global health problem affecting women, and to a lesser extent men, and constituted 25% of all cancers in 2012, with 883,000 cases each year in less developed and 794,000 cases in more developed regions of the world [1]. There were 458,000 breast cancer-related deaths worldwide (269,000 in developing and 189,500 in developed countries) in 2008 [2]. Furthermore, it is the leading cause of death in the world among women aged 40–59 years, and in premenopausal women, incidence and mortality has increased in South America, Central America and Africa [3, 4]. Analysis of the SEER (1988–2003 Surveillance, Epidemiology and End Results) Program data, comparing women less than 40 years old with those more than 40 years old, indicated that although they only constituted 6.4% of the study, they were more likely to die from breast cancer [5]. Limited breast health awareness and access to screening has a significant impact on the ability of health services to diagnose and treat the disease at a sufficiently early stage, and in many cases, by the time the patient presents at clinic, the cancers have already become aggressive. This problem is not unique to less developed countries as deaths also reached an all-time high during 2010 in the UK for women under 50 years old. The risks associated with breast cancer are well established and include genetic predisposition [6, 7], reproductive factors (menarche, parity, lactation, menopause) [8–10], environment (chemical exposures, drugs, infectious agents) [11, 12], demographic factors (age, race, sex, socio-economic status, geographic location) [13], systemic factors (epigenetics) [14] and lifestyle (smoking, alcohol use, diet, obesity, exercise) [15].

The major risk factor is age and the majority of breast cancer cases occur in women after menopause. However, the correlation of risk with reproductive, environmental and lifestyle parameters, strongly indicate the origins of many breast cancers are concealed by a prolonged period of dormancy that only manifest in later life [16]. Lifestyle parameters are unique among these factors, since they can be modified and consequently fall within the scope of the individual to proactively reduce these risks by taking preventative measures, whereas genetic, reproductive, environmental and demographic factors are beyond the control of most individuals. Women at highest risk, accounting for 5–10% of cases, are those who inherit gene mutations, and have 10- to 30-fold higher chance of developing breast cancer during their lifetime compared to the general population. Genetic predisposition can be identified initially through investigation of family history of the disease and confirmed by screening for predictive mutations associated with specific high-penetrance genes, such as the BRCA1, BRCA2, TP53 and PTEN genes [6].

The emergence of genomic technologies in the last 20 years has had a major impact on understanding disease stratification in breast cancer, which is not only providing insights on how the disease progresses, but clinical direction in targeted treatment and prediction of outcome. There are currently 5 main phenotypes, Luminal A, Luminal B, Her2, basal-like and Claudin-low, but further stratification can be anticipated [17]. At the simplest level, each phenotype is defined by expression or absence of three cell surface proteins, estrogen receptor (ER), progesterone receptor (PR) and human epidermal growth factor receptor (HER2). These phenotypes also correlate with normal mammary cellular development [18]. Claudin-low and Basal-like types, originating from breast stem and progenitor cells, respectively, constitute the majority of triple negative breast cancers (TNBC), which have a particularly poor prognosis, no targeted treatment, high chance of re-occurrence and poor overall survival. TNBC is also strongly associated with BRCA1/BRCA2 induced cancer [19] and with obesity in premenopausal women [20].

Because of extensive epidemiological and etiological studies, it is becoming easier to identify those women who are at highest risk of breast cancer. Unfortunately, none of these measures can predict when breast cancer will occur. Regular screening for the appearance of breast cancer, which is recommended for women reaching menopause, is even more critical in high risk women during their reproductive years. There are various screening modalities, each with advantages and disadvantages (Table 1), making them sub-optimal for more frequent monitoring as required by this group. As a consequence, the major means of preventative intervention for these women can be extreme, involving elective pre-symptomatic surgery to remove both breasts. Therefore, new and more sensitive approaches are required to detect the earliest stages of the disease, which will enable less drastic means of intervention. Initially, we review the various established screening methods, to understand their limitations.

**Table 1:** A semi-quantitative comparison of liquid biopsies that have been used for breast cancer biomarker discovery

Liquid biopsy	Volume	Accessibility	Discomfort	Reference sample*	Sample preparation	Biomarker concentration	Patient led collection	Convention
Plasma	++	+++	+++	X	++++	++	X	+++++
Urine	+++++	+++++	+	X	+++	+	√	++++
Saliva	+++	+++++	+	X	++	+	√	+++
Ductal lavage	+	+	++++	√	+	+++++	X	+
Random Periareolar Fine Needle Aspiration	+	+	+++++	√	++	+++++	X	+
Nipple Aspirate Fluid	+	+++(+)	++	√	+	+++++	√	++
+	Small	Invasive	No discomfort/no distress		Extensive processing required	Low		Infrequent use for biomarkers
+++++	Large	Easily available	Painful/stressful		Limited process required	High		Frequent use for biomarkers

(+) Accessibility enhanced by oxytocin nasal spray

*Comparison of healthy and disease sample pairs provides an internal reference

## Breast cancer detection

### Self-examination

Self-examination encourages women to be involved in active surveillance of their own breast health, increasing awareness of changes, both natural and abnormal. It provides an effective way for early detection of up to 50% tumours and improves discovery of other breast-related diseases without involving specialist equipment or professional health care assistance [21]. However, the prevalence of breast self-examination is still low, particularly in educationally-deprived and developing countries [22].

### Mammography

Mammography is by far the most common approach for screening breast cancer. In Europe, women aged 50–70 are invited for breast screening every 2–3 years, while in US annual mammograms are recommended from 50 to 74 years equating to nearly 39 million images in 2014. Women who have an increased risk of developing breast cancer, but are too young to join national screening programs, are offered annual screening using mammography and magnetic resonance imaging (MRI) based on a risk–benefit decision. Sensitivity of mammography can vary from almost 100–40% dependent on tissue composition [23, 24], and positive association for prediction of disease is only 50%. False-positive rates in breast cancer screening are a significant limitation, as high call-back rates and unnecessary biopsies result in increased cost, radiation exposure, and patient anxiety during re-screening [25, 26]. Over a period of 20 years, based on 7 trials of more than 600,000 women, screening was shown to reduce breast cancer mortality by 15% however over-diagnosis was estimated at 29% [27, 28]. In particular, mammography is approximately 10–15% less sensitive in women under 50 than in postmenopausal women [29]. Women in their 40s screened within the UK Age Trial had a 5% risk of a false-positive result at their first screen [30] and a random clinical trial of 161,000 women showed that women aged 39 onwards did not significantly reduce their risk of dying from breast cancer within 5–15 years [31].

The continuing development of mammography, including digitisation with improved resolution (full field digital mammography), has strengthened its position as the Gold standard for screening but still requires the presence of a substantial mass in the breast for a diagnosis [32, 33]. Many breast tumours may have already metastasised before detection by mammography. Equally, a mammogram cannot always distinguish between benign micro-calcifications associated with low risk DCIS (therefore may not require surgical treatment) and higher risk DCIS that will progress to a malignant invasive tumour [34]. Even when supported by histopathological screening of core biopsies, there has been a tendency to over-diagnose and over-treat DCIS cases [35]. Consequentially, some women receive a false-positive diagnosis resulting in unnecessary surgery to remove the lump or whole breast. Radiation exposure has also been shown to contribute to increased incidence of breast cancer, particularly in high risk populations, resulting in heart disease induced by breast or cell wall radiotherapy [23].

One of the challenges for imaging methods is breast tissue density, which is higher in younger women, making their mammograms harder to interpret, whereas postmenopausal women with extensive fatty breast tissue or ductal atrophy are more likely to have an accurate diagnosis [36]. Breast density is strongly associated with tissue composition (collagen, epithelial cell and non-epithelial cell content, glandular area), genetic influences and hormonal regulation (prolactin, IGF-1). Furthermore, mammographic density is positively associated with alcohol consumption, smoking in post-menopausal women and with breast cancer [36, 37]. It is possible to quantitatively measure breast density using established and specialised imaging modalities in combination with bespoke algorithms [38], however this strategy has not been widely accepted.

### Other imaging techniques

Digital breast tomosynthesis (DBT) is a relatively new screening approach, which uses an X-ray beam in an arc around the breast to provide a 3D reconstruction of the tissue. In a comparison of DBT with digital mammography for 2666 breast lesions, the former had improved performance (sensitivity and specificity of 90 and 79%, respectively) compared to the latter (89 and 72%, respectively) [39], but was also subject to the same limitations incurred by breast density. There are fewer recalls with DBT compared to digital mammography, particularly in younger women, and although approved by the Food and Drug Administration for breast screening, it is not widely available [40]. Ultrasonography, using high-resolution linear transducers, is readily accessible, similar in cost to mammography and moderately improves cancer detection, but has lower specificity, lower positive predictive value and was similarly obfuscated by tissue density [41].

Contrast-enhanced MRI, by comparison is not limited by breast density, nor does it use ionising radiation. In a review of 11 studies comparing MRI with digital mammography, the former achieved 94% sensitivity, but identified few cases of cancer in addition to those discovered by digital mammography alone [42]. Furthermore, MRI exhibited relatively low specificity, was time consuming, had limited accessibility and high cost to run. Positron Emission Tomography with computer tomography (PET-CT) uses gamma-radiated emitting fluorine-18 fluorodeoxyglucose (FDG), a glucose analogue, injected into arm veins to accumulate in areas of high metabolic activity, such as tumours, and is then detected using a PET-CT x-ray scanner [43]. Sensitivity of PET-CT ranged from 71 to 90%, which was improved further when combined with mammography, but results were affected by breast size [44]. However, PET-CT is more often used to assist surgery to remove diseased tissue rather than as a front line routine screen for initial detection of breast cancer. Wave elastography uses ultrasound or MRI techniques to measure tissue stiffness, which is positively associated with a tumour mass compared to normal tissue. The application of shear wave elastography, which uses the force of acoustic radiation produced by an ultrasound beam, demonstrated improved sensitivity and specificity [45]. Electrical impedance scanning is another modality under development for breast cancer detection especially in young women with dense breasts, which is based on lower electrical impedance in malignant tumours compared to the surrounding normal tissue [46]. Recent technological advances have facilitated dynamic thermal analysis of the breast by recording of circadian rhythm variations and analysing the recorded data using highly sophisticated statistical algorithms, but this approach currently suffers from high false positive rates [47]. Although showing great promise, none of these innovative imaging approaches are in a position to replace mammography as the mainstream approach to routinely detect breast cancer and most importantly cannot differentiate between benign and malignant growths.

## Biomarkers

There have been extensive studies to identify breast cancer biomarkers, but with varying degrees of success. Genomics has already stratified the disease to identify high risk individuals and histopathological approaches are used to determine ER, PR and HER2 expression, helping to direct clinical intervention. Indeed, microarray-based technologies for routine prognostic screening of multigene signatures (for example, 70-gene MammaPrint for tumour aggressiveness/chemotherapy requirement/metastatic prognosis [48], Celera 14-gene for metastasis score [49], Oncotype-DX 21-gene signature for measuring risk of re-occurrence [50], and 76-gene Veridex signature for tamoxifen therapy benefit [51]) have been invaluable in supporting treatment of ER/PR positive breast cancers. However, the identification of a specific biomarker for the detection of breast cancer at the earliest stage that can be analysed in biopsies, has so far evaded the diagnostic industry. Proteins and metabolites have been discovered which were increased in malignant tissues compared to normal, but were subsequently found to be diluted beyond the level of detection in plasma or urine, or were found not to be specific for breast cancer. These have been reviewed extensively elsewhere and will not be covered here [52]. In addition, depletion methods may be required to remove the most abundant proteins such as albumins and immunoglobulins from plasma, prior to analysis, which can result in losses and further reduced sensitivity. It is therefore not surprising that a comprehensive review of breast cancer biomarkers in 2007 by the Update Committee of the American Society of Clinical Oncology failed to recommend any of the most promising candidates, including blood levels of CA 15-3 and CA 27.29 (both forms of mucin-1) for diagnosis, detection of recurrence, decisions on therapy or metastasis, or circulating truncated extracellular HER2 for detection of breast cancer [53]. Clearly, accessing tissue biopsies to look at tumour-associated biomarkers, where they are most concentrated, on a regular basis is not practical, though most of the accepted biomarkers ER, PR, HER2, urokinase-type plasminogen activator (uPA) and plasminogen activator inhibitor 1 (PAI-1) are currently analysed in tissues by immunohistochemistry or ELISA [53]. Hence, the challenge remains, how to provide a low cost, safe, simple, sensitive and specific method for detecting breast cancer, early enough, avoid unnecessary overtreatment and surgery? In this context, molecular diagnostic approaches for the early detection of breast cancer remain largely untapped.

## Breast-specific liquid biopsies

By far the best liquid biopsies (or biofluids) for developing a screening diagnostic are those readily accessible and in close proximity to the disease area, such as saliva for oral cancers or urine for bladder or renal cancer (Table 2). In this respect, various methods have been developed to access ductal liquids that are associated with cells that are the origin of the majority of breast cancers. We present, a case for using nipple aspirate fluid (NAF) for routine breast cancer screening, but initially, review the various breast-associated fluids that have been studied, with a specific emphasis on proteomics characterisation.

**Table 2:** A synopsis of the established approaches for the detection of breast cancer

Modality	Indication	Sensitivity (%)	Specificity (%)	Advantages	Disadvantages	References
Self-examination	Tumour detection	53.90	54–59	A free and easy way to look for cancer and reduce mortality	Some breast lumps can be missed, cause unnecessary distress	[21]
Mammography	Tumour detection	73–86	88–93	Inexpensive, highly portable and does not necessarily require a contrast agent	Discomfort, limited depth penetration, challenging spatial localization, and radiation exposure, False positive and false negative results	[29]
Ultrasound, especially with contrast enhancement	Detection tumour characterization	61.40	82	Highly portable, inexpensive molecular microbubble agents possible	Operator dependence contrast agents confined to vascular system	[41]
Magnetic resonance imaging (MRI)	Tumour characterization	77–99	81–99	Quantification of tumour perfusion and tumour capillary permeability	Confined space, contrast design limited by need for magnetic atom	[42]
Positron emission tomography	Detection response evaluation characterization	64–96	73–99	Wide range of molecular imaging probes Tracer imaging without perturbing biologic system	Limited spatial resolution (improved with use of non-contrast computed tomography), some radiation exposure	[43]
Histopathology	Detection, tumour characterization	90	88	Differentiating benign and malignant	Discomfort and painful because a surgical procedures required, risk of complications such as infection and bleeding. Can result in over-diagnosis and overtreatment	[35]

## Milk and colostrum

There have been a number of proteomics studies on milk purely from a functional perspective, unrelated to cancer, with a recent study identifying up to 1600 proteins [54, 55]. Colostrum, produced 2 or 3 days prior to lactation, preparing the infants digestive system for milk as a food source and immunisation against infection, has also been characterised. In a study of 100 samples using 2D liquid chromatography mass spectrometry (LC MS), 151 proteins were identified after immunodepletion (to remove the most abundant proteins), including 83 found in colostrum but not milk [56]. Although available in useful volumes for analysis, the period of production during the reproductive phase of life is relatively narrow and in most cases will not overlap with breast cancer development. Consequently, there has been very little research on proteomic profiling of breast cancer in milk or colostrum because of the relatively small proportion of women in which the disease and postnatal breast-feeding, coincide. Nevertheless, Schneider et al. were able to profile samples from a small cohort and identified proteins that were uniquely present in milk from women diagnosed with breast cancer [57].

## Breast cyst fluid

Several epidemiological and prospective studies indicate there may be a relationship between cystic breast disease and cancer [58]. There are two types of breast cyst (Type I or apocrine cysts and Type II) differentiated on the basis of morphological and cellular characteristics. Apocrine cysts differ from Type II cysts in having a higher K^+^/Na^+^ ratios while Type I cysts are more strongly related with breast cancer [59]. According to Mannello et al., more than 100 studies have identified 81 proteins in breast cyst fluid, with the major components identified as albumin, prolactin inducible protein, Zn-α_2_-glycoprotein, and apolipoprotein D [59]. A 2-D gel electrophoresis/mass spectrometry study on apocrine macrocyst fluid identified that 15-hydroxy-prostaglandin dehydrogenase and 3-hydroxymethylglutaryl-CoA synthase were associated with cysts and tumour tissue and were absent in matched normal tissue [58].

## Ductal lavage (DL)

Ductal lavage (DL) is a non-surgical breast epithelial sampling procedure that was developed to identify high risk breast cancer women and to detect malignant lesions in breast epithelial cells. In the DL procedure a micro-catheter is cannulated to the ducts, infused by a saline solution, and then aspirated by a suction device to collect cells from the lining of ducts. More cells are acquired through ductal lavage than from nipple aspiration [60], and liquid biopsies can be obtained from women that do not yield fluid by passive nipple aspiration or discharge or from breast massage. Ductal lavage containing sufficient cells was collected from 31 women (out of 36 volunteers) diagnosed with breast cancer and analysed for atypical cytology as a possible diagnostic indicator, however only 13% produced a significant positive indication [61]. A separate study of 30 samples found that 23.3% of women had atypical lavage cytopathology; interestingly these women had normal mammogram screening of breast, indicating the potential for improved sensitivity [62]. The isolation of cells from ductal lavage opens up the opportunity for applying molecular biology approaches. Multiplex methylation-specific polymerase chain reaction (QM-PCR) was used to quantitate cumulative gene promoter hypermethylation in multiple genes, which are markers for breast cancer, and found to double the sensitivity of detection of cancer cells compared with cytology [63]. An attempt to identify biomarkers of tamoxifen action (e.g. changes in expression of estrogen receptor α, Ki-67 and cyclooxygenase-2) in ductal lavage, however, found no significant cytological or molecular variations in patients [64]. Recently, an improved method of ductal aspiration, collecting multiple aliquots, considerably increased the cell recovery with 45/50 subjects yielding more than 1000 cells and 50% of those producing more than 20,000 cells with 80–100% epithelial cell purity. This provided genomic DNA, RNA and miRNA samples for analysis however, to date only qualitative observations of the molecular profiles have been reported [65]. Do Canto et al. identified more than 700 miRNAs from ductal lavage from women with unilateral breast cancer, of which 17 were differentially expressed between tumour and paired normal samples and have previously been associated with tumorigenic processes and signalling pathways for invasiveness and metastases [66]. In a related study, the metabolomic profiles of ductal lavage of 43 women with breast cancer was acquired [67]. From a total of 2098 compounds (detected in both positive and negative ion mode with a QTOF mass spectrometer), a signature of 21 metabolites (including *N*-acetyl-DL-tryptophan, *N*-linoleoyl taurine, *trans*-2-dodecenoylcarnitine and specific phospholipid isoforms) was determined to provide a ROC Curve of 90.7% sensitivity in diagnosing breast cancer. However, ductal lavage can cause considerable discomfort which has prevented widespread clinical use [68]. Furthermore, the process of flushing the ducts, results in dilution of the protein components and hence reduced sensitivity for biomarker profiling.

## Random Peri-areolar Fine Needle Aspiration (RPFNA)

RPFNA, developed by Dr. Carol Fabian in 1998, provides a snap-shot of the breast by sampling cells from the entire breast of asymptomatic women [69, 70]. The major advantage of RPFNA is that it can be performed in the majority of women and the cell yields vary from 72 to 85%, considerably higher than ductal lavage. [71]. After anesthetizing the breast with 1% lidocaine, five needle aspirations are made on the lateral breast site and four from the middle skin of each. The aspirated fluid consists of epithelial, immune, stromal and adipose cells [70].

A clinical trial of 480 women indicated that RPFNA increased detection of cytological atypia associated with breast cancer in high-risk women (based on family history, a prior diagnosis and precancerous biopsy) [70]. Of the cohort, 20 women subsequently developed breast cancer after 45 months (7 DCIS and 13 invasive), indicating a promising potential for very early diagnosis. RPFNA was used for a chemoprevention study of alpha-difluoromethylornithine (DFMO) in 119 high-risk women, but found no change in cytology or other RPFNA-based molecular markers such as expression of proliferating cell nuclear antigen, p53 or epidermal growth factor receptor [72]. A proteomic microarray study found that up to 60 phosphoproteins can be verified in triplicate from 5000 to 10,000 micro-dissected RPFNA epithelial cells, suggesting the possibility to track signalling pathways in order to understand the molecular changes occurring in mammary carcinogenesis [73]. The heterogeneous nature of the cell populations being tested for specific molecular markers and considerable discomfort to obtain the samples, are key limitations of the RPFNA approach. Furthermore, the difficulty in reproducing the method may preclude a role in screening of high-risk women that involves repeated harvesting of material.

## Nipple aspirate fluid (NAF)

The breasts of adult non-lactating women secrete small volume of fluid, called “nipple aspirate fluid” (NAF, which here also includes spontaneous nipple discharge) into the breast ducts [74]. The research presented within this section relates to nipple secreted fluids collected by non-invasive methods, either passive discharge or by use of massage or pumps, differentiating them from the invasive approaches previously described (ductal lavage RPFNA, etc.). There have been a series of seminal reviews outlining the importance of NAF for diagnosing breast cancer [75–78], however, the current review aims to promote the progression that has been achieved through the application of ‘omics’ strategies. The fluid passes down the main ducts and ampullae through alveolar glands of breast, from which it enters the lymphatic and blood circulation [79]. Under normal conditions, the breast fluid cannot escape from the nipple because the ducts are blocked by viscous and dried-up secretions or because of the presence of constrictive bands of smooth muscle and keratinized epithelium [79]. To maintain stable physiology of the breast, an equilibrium exists between fluid secretion and re-absorption. Several factors are associated with NAF production; age, ethnicity, early menarche, history of lactation, high dietary fat consumption and dietary intake of lactose [80]. There is also a direct relationship between ear wax and NAF because both are produced by modified apocrine glands (ceruminous and mammary, respectively). Women with wet ear wax yield more NAF, compared to women with dry ear wax [81]. Premenopausal women with lactation experience, aged 30–50 years and who had early onset of menarche produce more NAF compared to those who have not had children [80]. A study of 25 to 49-year-old premenopausal nulliparous women found that proportionately, Asian-descendant women were less likely to produce NAF compared to White American women [53].

NAF collection has been achieved with varying degrees of success dependent on the method and the practitioner and, in some cases, has deterred researchers from further investigation. Electronic and manual breast pumps (normally used for lactation), massage, warming and combinations of each have been used to acquire NAF samples [82]. Most promising has been the use of oxytocin nasal spray which helps the release of already existing fluid in the ducts increasing collection in 95% of patients and volunteers [83].

NAF is a rich source of molecular and cellular information. Indeed, in a composite study of published data on NAF cell content, cellularity or proliferative epithelial disease was observed to be an independent risk determinant for breast cancer development [84]. This reflects the increased exfoliation of epithelial cells lining the ducts and lobules as they proliferate through hyperplasia, in situ carcinoma and invasive carcinoma with disease progression [85]. The released cells not only increase in number but also change in appearance, exhibiting morphological differences (irregularly shaped nuclei, rough endoplasmic cisternae and well-developed Golgi complexes associated with nucleic acid and protein synthesis) [86].

NAF is also composed of a variety of endogenous substances such as lactose, proteins, fatty acids, hormones (estrogens, androgens, progesterone), sterols, but may also contain exogenous substances such as nicotine and cotinine from cigarette-smoking [74]. The colour of NAF varies from clear to brown, bloody, black, pale yellow, dark yellow, white or green [87], and is associated with the concentration of cholesterol, estradiol, estrone, cholesterol epoxides and peroxidated lipids [88]. NAF has also been shown to contain microorganisms and the ductal microbiome was distinctly different from that of the nipple and areolar skin [89]. In addition, a comparison of 6 breast cancer patients (contralateral sample) with 6 healthy controls (both breasts) showed variations in particular microbe genus incidence, indicating a potential role in the disease.

The colour of NAF is more an epidemiological factor than an indicator of the risk of breast cancer, however one study found that women have a higher risk of breast cancer with bloody or brown nipple discharge, compared to those which were white, cream, yellow or green [90]. Another study of 327 women found that the frequency of red or brown colour was increased with progression of disease from pre-cancer to cancer and surgical biopsy has more influence on NAF colour compared to needle biopsy [91].

NAF production, nutritional aspects and estrogen level have been found to be related to breast cancer risk. A large scale study of 1496 participants (1347 white and 153 black women) found a positive association between higher dietary fat and NAF secretion in the group aged 30–44 years [92]. As obesity is associated with a high fat diet and is a major risk factor for breast cancer, correlation of fat intake, and NAF production and composition may be helpful for breast cancer prevention and prognosis [93]. A link between lactose and soy intake has also been reported, however contrary results from a randomized crossover trial discovered no influence of soy on NAF volume and circulating estrogen level [94]. On the other hand, a fruit-and-vegetable diet was inversely related with NAF production while decreasing the circulating hormone concentration [95]. The concentration of micro-nutrients, such as carotenoids and soy isoflavones in NAF, was also related to dietary intake [96].

## NAF biomarkers

In order to place the ‘omics’ approaches to global analysis of molecular events in NAF profiling, into perspective, it is important to appraise the extensive research on specific biomarker targets that has been undertaken in NAF (Table 3). Based on the differential levels of testosterone in serum from pre- and postmenopausal women, Sauter et al. measured testosterone levels in NAF samples and found it would be a suitable biomarker to predict breast cancer risk [97]. A separate study, which measured the level of free and albumin-bound testosterone in NAF, found high levels of the former in premenopausal women with breast cancer [98].

**Table 3:** A summary of putative biomarkers and their biological function that have been identified in nipple aspirate fluid

Biomarker (s)	Characteristics	References
Prostate specific antigen (PSA)	Inversely proportional to disease stage, size of tumour, node status and distant metastases	[102–104]
Thomsen–Friedenreich (TFr) and Tn antigens	Predictive for the presence of breast cancer or atypia	[99, 100]
Testosterone	Predictive in postmenopausal women only	[97, 98]
Superoxide dismutase (SOD-1)	Involved in cancer initiation and progression by ROS related damages	[105]
Protein DJ-1	mRNA level increased but protein level decreased in tissue	[106]
Cytokines/chemokines	High level of pro-inflammatory C–C and CXC chemokines.	[107]
Plasminogen activator inhibitor-1 (PAI-1), urokinase-type plasminogen activator (uPA)	Promotes breast cancer invasion and metastasis	[110, 111]
Serotransferrin protein (TF) and ferritin (FTN)	Proliferation of cancer cells	[109]
C-reactive protein (CRP)	Serum biomarker for metastasis of different type of cancers	[112, 113]

Thomsen–Friedenreich (TFr) and Tn antigens are aberrant O-linked mono- (GalNAc) or disaccharides (Gal-GalNAc) found on cell surface glycoproteins and associated with the progression of numerous cancers including bowel, skin, prostate and breast. A study of the expression of TFr and Tn antigens found that NAF of 90% breast cancer patients have high content of TFr and Tn compared to normal NAF, because both antigens are present on the surface of epithelial cancer cells and lipids [99]. A recent study of 137 women found that the concentrations of TFr and Tn antigens were lower in women with benign disease, compared to those with atypical hyperplasia. They also found that the expression of TFr is more predictive for the presence of breast cancer or atypia compared to Tn [100].

Proteins are major constituents of NAF with concentrations that can be higher than plasma, up to 170 mg/ml. NAF, most importantly, is enriched for proteins originating from epithelial cells lining the duct [101], some of which have been evaluated as potential biomarkers of breast cancer. Prostate specific antigen (PSA), also known as kallikrein-3 (KLK3), first identified in seminal plasma and prostatic tissue, produced by the epithelial cells lining the acini and ducts of prostate gland, has also been identified in female breast tumours [102]. A study of NAF found that women with no risk factors or family history of breast cancer had high levels of PSA, but women with precancerous or invasive cancer had reduced levels [103]. Furthermore, PSA levels were inversely proportional not only to disease stage, but also tumour size, node status and distant metastases [104].

The concentration of superoxide dismutase [Cu–Zn] (SOD-1) in NAF was decreased in breast cancer patients compared to healthy individuals [105]. SOD-1 is involved in cancer initiation and progression caused by reactive oxygen species-related damage. Therefore, it was proposed that measuring the concentration of SOD-1, a key antioxidant enzyme in breast microenvironment, may be helpful to differentiate between the normal and tumour breast.

The expression of anti-oxidant oncogene DJ-1 mRNA is increased in ductal carcinoma tissues but the opposite effect was observed at the protein level, where expression is decreased and contrarily was elevated in blood of breast cancer patients. A study on NAF collected from 136 patients identified high levels of DJ-1 protein in NAF from breast cancer patients, but low levels in benign papilloma cases [106].

NAF samples collected from non-cancer and cancer women for cytokine profiling found no difference in anti-inflammatory cytokines (IL-4, IL-9, IL-10 and IL-13), pro-inflammatory cytokines (IL-2 and interferon-γ), immuno-modulatory interleukins (IL-5, IL-7) or chemokines (RANTES, IP-10, eotaxin). However, NAFs from cancer patients with high levels of aluminium in the breast microenvironment, had higher concentrations of pro-inflammatory cytokines (IL-1β, IL-6, IL-12 p70, and TNF-α), and C–C (MCP-1 and MIP-1α) and CXC-type chemokines (IL-8) compared to those cancer patients with low aluminium levels. This indicated a significant correlation between pro-inflammatory cytokines (IL-6), monocyte/macrophage chemo-attractant chemokines (MIP-1α and MCP-1), oxidative stress and aluminium content in cancerous NAFs [107].

Cancer cells contain high levels of transferrin (TF) and ferritin (FTN), as well as higher expression of TF receptors compared to normal cells, suggesting proteins involved in Fe metabolism play important role in the proliferation of breast cancer cells [108]. A study of NAF collected from 66 women found that cancer patients (particularly postmenopausal) have high levels of TF and FTN, compared to healthy women [109]. Hence, measuring soluble FTN and TF in NAF may help the early identification of women with increased breast cancer risk, even though these proteins are not expressed by local breast tissues.

Plasminogen activator inhibitor-1 (PAI-1), normally found in plasma, promotes breast cancer invasion and metastasis by directly inhibiting proteases, suggesting that excessive plasmin proteolysis may inhibit the assembly of tumour blood vessels, modulation of cell adhesion and the stimulation of cell proliferation [110]. Significantly higher levels of uPA and PAI-1, along with the Thomsen–Friedenreich antigen, have been reported in NAF of women with cancer, with the former more predictive for postmenopausal and the latter more indicative for premenopausal patients [111].

C-reactive protein (CRP) is already a developed serum biomarker for metastasis of various cancers including advanced stages of breast cancer [112]. Elevated CRP levels in ductal epithelia of the breast are an indicator of inflammatory processes associated with an early benign stage. A study on 59 samples found that CRP in NAF was positively related to the Gail model for breast cancer risk [113].

## NAF proteomics

Proteomics approaches for characterising NAF has previously been reviewed by Mannello et al. [114], since which there has been rapid advancement in mass spectrometric technology and the development of new quantitative strategies. On the basis of the unique characteristics of NAF, proteomic analysis should serve as a useful approach to understand the physiology of breast cancer and for biomarker discovery. However, early proteomic profiling of NAF samples collected from cancerous and non-cancerous breasts of patients using surface-enhanced laser desorption ionization mass spectrometry (SELDI-MS), revealed no significant differences in the SELDI-MS peak profiles [115]. Use of more powerful separation techniques started to reveal differences. Varnum et al. identified 64 proteins in immune-depleted NAF samples, using an ion trap mass spectrometer, among which 15 had previously been reported to be altered in tumour tissue and serum from women with breast cancer, including osteopontin and cathepsin D [116]. Two-dimensional PAGE separation of proteins, followed by in-gel digestion with trypsin and matrix-assisted laser desorption ionization time-of-flight mass spectrometer (MALDI-TOF) analysis, identified 41 components in NAF [117]. Among these, levels of prolactin-inducible protein, apolipoprotein D, and α_1_-acid glycoprotein, were observed to be changed in cancer NAF samples. Further validation by ELISA, indicated that expression of these proteins correlated with pre-/postmenopausal status and cancer stage. Pawlik et al. [118] used Isotope-coded affinity tag (ICAT) tandem mass spectrometry (MS) for qualitative and quantitative analysis of tumour specific proteins in NAF, identified 353 peptides from 39 proteins in NAF samples from 18 women with breast cancer and 4 healthy volunteers. Alpha-2-HS-glyoprotein, was found to be decreased, whereas lipophilin B, beta-globin, hemopexin and vitamin-D binding protein were increased in breast cancer NAF samples. A recent study on six NAF samples (3 healthy individuals and 3 cancer patients) analysed by using an LTQ-Orbitrap XL mass spectrometer, identified more than 854 unique proteins, including established putative breast cancer biomarkers candidates, cancer antigen 15.3, tissue plasminogen activator, uPA, and cathepsin D [119]. Recently, in a series of experiments to optimise protein separation from a NAF sample, Brunoro et al. identified 557 different proteins [120]. The most complete proteomics study was performed by Shaheed et al. [121], identifying more than 1900 unique gene products including mitogenic growth factors (IGF1, IGF2, EGF, PDGFC, PGGFD, TGFβ1, VEGFA), cell adhesion proteins (CEACAMs, NCAM2, ICAM1), established breast cancer biomarkers (EGFR, mucin-1/CA 15-3, mucin-16/CA-125, MUCL1, cytokeratins 5, 8, 14 and 18) as well as 46 candidate biomarkers under investigation by the National Cancer Institute Early Detection Research Network [122]. A comparison of matched NAF pairs, from a healthy volunteer and patients with benign, DCIS or invasive carcinoma, detected an average of more than 1200 proteins per sample [121]. While matched pairs exhibited strong similarity in profile, individuals showed significant differences, confirming the observations by Brunoro et al., using SDS PAGE and 2D-DIGE analysis of NAF [123]. Indeed, the composition of the healthy volunteer samples were disproportionately high in milk proteins, despite the fact that the subject had never breast fed. In this case, milk proteins were diagnostic for galactorrhea caused by prescribed medication inducing a nipple discharge [121]. The different protein profiles identified in NAF samples, collected by different groups, clearly highlight the potential for identifying biomarkers that could be related to the early development of breast cancer.

## Conclusions

One of the major causes of death among women throughout the world is breast cancer. Despite tremendous progress in understanding the causes and advances in treatment regimens, the options for women at high risk of breast cancer are limited to drastic surgical intervention, because current methods for regular screening have significant limitations. Identifying biomarkers indicative of the earliest stages of malignancy has great potential, but so far it has not been fully explored. Cancer associated tissues are not readily accessible on a regular basis and plasma dilutes biomarkers once released from the diseased area. There would be huge benefits in analysing a biofluid collected from healthy volunteers and breast cancer patients that directly originates from the affected organ source. In this respect, NAF-based biomarkers offer great potential for developing an innovative non-invasive, patient-led screening strategy. NAF has multiple advantages as a liquid biopsy for detection of breast cancer: (1) premenopausal women, for whom current diagnostic modalities are limiting, are more likely to produce NAF than postmenopausal women where ductal atrophy is prevalent, (2) NAF production is non-invasive and causes less discomfort compared to other breast cancer screening procedures [124–126], (3) obtaining matched pairs of samples provides an “internal” control for comparing disease with healthy, (4) NAF is produced in close proximity to the cells lining the ducts, which are associated with 85% of all breast malignancies, and as a consequence is symptomatic of breast health, (5) biomarkers remain highly concentrated for analysis (compared to blood and urine where massive dilution significantly reduce detection sensitivity for tissue-derived proteins), and (6) sample preparation is reduced compared to tissues, which require yield-reducing protein extraction steps. NAF volumes are small, but protein concentrations are very high and are more than adequate for replicate analyses with state-of-the-art mass spectrometric techniques.

**Fig. 1:**
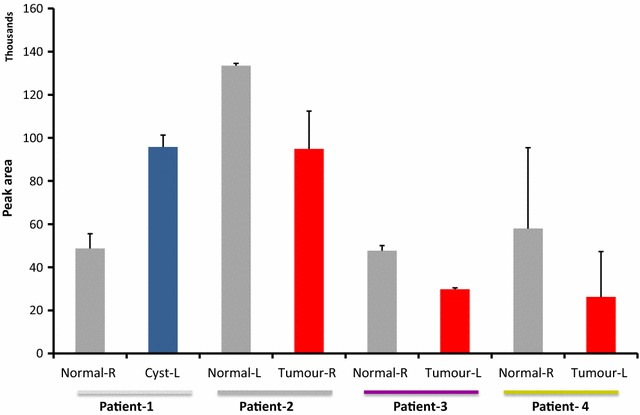
Multiple reaction monitoring mass spectrometry analysis (n = 3) of a proteotypic peptide of prolactin inducible protein in matched NAF samples from four patients

**Fig. 2:**
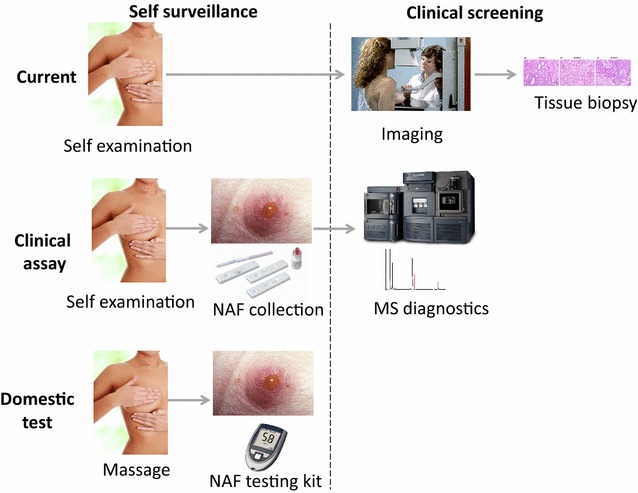
Paradigm shift in breast cancer detection

In a study of matched pairs of NAF samples by multiple reaction monitoring mass spectrometry (MRM MS), significant differences in prolactin inducible protein were observed between the diseased breast and the contralateral healthy breast (Fig. 1). With an MRM MS approach there is the potential to develop a multiplexed assay that measures a number of markers of breast health, defining patient-specific composition, that can be monitored in a longitudinal study. A wider study of a panel of biomarkers will provide increased specificity and would enable development of a clinical assay. This would provide high-risk women with a safe, convenient breast cancer detection program, which could be applied regularly to breast health surveillance, detect the earliest stages of the disease and avoid extreme preventative intervention procedures, such as elective bilateral mastectomy. Ultimately, detection of biomarkers in NAF could represent a paradigm shift in breast cancer management empowering women to express samples at home on a monthly or quarterly basis and analyse their own samples with a diagnostic kit, massively reducing the burden on health services (Fig. 2).

## Abbreviations

CRP: C-reactive
DBL: digital breast tomosynthesis
DCIS: ductal carcinoma in situ
DIGE: difference gel electrophoresis
DL: ductal lavage
ER: estrogen receptor
FDG: fluorodeoxyglucose
FTN: ferritin
HER2: human epidermal growth factor receptor 2
LC MS: 2D liquid chromatography mass spectrometry
MRI: magnetic resonance imaging
MRM MS: multiple reaction monitoring mass spectrometry
NAF: nipple aspirate fluid
PAI-1: plasminogen activator inhibitor 1
PET-CT: positron emission tomography with computer tomography
PR: progesterone receptor
PSA: prostate specific antigen
QTOF: quadrupole time-of-flight
ROC: receiver operating characteristics
RPFNA: Random Peri-areolar Fine Needle Aspiration
SOD-1: superoxide dismutase [Cu–Zn]
TF: transferrin
TFr: Thomsen–Friedenreich antigen
TNBC: triple negative breast cancers
uPA: urokinase-type plasminogen activator
